# The role of microglia in prion diseases and possible therapeutic targets: a literature review

**DOI:** 10.1080/19336896.2021.1991771

**Published:** 2021-11-09

**Authors:** Ananda Sampaio Lamenha Falcão de Melo, Juliana Louise Dias Lima, Maria Carolina Silva Malta, Natália França Marroquim, Álvaro Rivelli Moreira, Isabelle de Almeida Ladeia, Fabrizio dos Santos Cardoso, Daniel Buzaglo Gonçalves, Bruna Guimarães Dutra, Júlio César Claudino dos Santos

**Affiliations:** a Faculdade de Medicina, Universidade Federal de Alagoas, Alagoas, AL, Brazil; b Department of Neurology, Centro Universitário Governador Ozanam Coelho, UniFacog, Ubá, MG, Brazil; c Núcleo de Pesquisas Tecnológicas, Universidade De Mogi Das Cruzes, Mogi das Cruzes, SP, Brazil; d Department of Psychology and Institute for Neuroscience, University of Texas (Ut), Austin, TX, USA; e Universidade Federal do Amazonas, Ubá, AM, Brazil; f Laboratório de Neurociências, Departamento De Neurologia E Neurocirurgia, Universidade Federal de São Paulo, São Paulo, Sp, Brazil

**Keywords:** Creutzfeldt-Jakob disease, microglia, neuroinflammation pathways

## Abstract

Creutzfeldt-Jakob disease (CJD) is a rare and fatal condition that leads to progressive neurodegeneration due to gliosis, vacuolation of central nervous system tissue, and loss of neurons. Microglia play a crucial role in maintaining Central Nervous System (CNS) homoeostasis, both in health and disease, through phagocytosis and cytokine production. In the context of CJD, the immunomodulatory function of microglia turns it into a cell of particular interest. Microglia would be activated by infectious prion proteins, initially acquiring a phagocytic and anti-inflammatory profile (M2), and producing cytokines such as IL-4, IL-10, and TGF-β. Therefore, microglia are seen as a key target for the development of new treatment approaches, with many emerging strategies to guide it towards a beneficial role upon neuroinflammation, by manipulating its metabolic pathways. In such a setting, many cellular targets in microglia that can be involved in phenotype modulation, such as membrane receptors, have been identified and pointed out as possible targets for further experiments and therapeutic approaches. In this article, we review the major findings about the role of microglia in CJD, including its relationship to some risk factors associated with the development of the disease. Furthermore, considering its central role in neural immunity, we explore microglial connection with other elements of the immune system and cell signalling, such as inflammasomes, the complement and purinergic systems, and the latest finding strategies to guide these cells from harmful to beneficial roles.

## Background

Creutzfeldt-Jakob disease (CJD) is a rare and fatal condition that leads to progressive neurodegeneration due to gliosis, vacuolation of central nervous system tissue, and loss of neurons[1].It is a human type of transmissible spongiform encephalopathy and it is classified into four different phenotypes, the most common being the sporadic CJD (sCJD) – characterized by the absence of previous risk factors[2]. Although some defend the role of an agent with a nucleic acid genome as a possible aetiology, the accumulation of misfolded forms of cellular prion protein (PrP^C^), known as infectious prion proteins (PrP^Sc^), is a well-established mechanism of disease development[3].

Microglia are the main immune figure of the central nervous system (CNS), coming from the yolk sac as primitive macrophages that seed the CNS before birth and persist there throughout adulthood[4]. They play a crucial role in maintaining CNS homoeostasis, both in health – by contributing to neurogenesis and synaptic formation – and disease – through phagocytosis and cytokine production. It is well known that microglia are the main neural cells in the context of neuroinflammatory conditions, since they can modulate all phases of inflammation – positively or negatively – through their polarization profiles: M1 and M2[5].

In the context of Creutzfeldt-Jakob disease, the immunomodulatory function of microglia turns it into a cell of particular interest. Microglia would be activated by PrP^Sc^, initially acquiring a phagocytic and anti-inflammatory profile (M2), and producing cytokines such as IL-4, IL-10, and TGF-β[6]. However, some argue that, once phagocytosis is not enough, accumulation of PrP^Sc^ leads to neuronal damage and guides microglia towards a proinflammatory archetype (M1), with the secretion of TNF-α, IL-1β and, IL-6. These cytokines intensify the parenchyma destruction and also turn astrocytes into a deleterious A1 cell type[5]. However, recent studies have pointed out that microglial activation is multidimensional and varies significantly depending on the context, with these cells rarely assuming a complete M1 or M2 phenotype[5]. It is expected that current studies discover more activated microglial states, which could help understand better how microglia lose their homoeostatic behaviour in neurodegenerative disease and also how to more selectively interfere with this process, slowing disease progression of disorders like CJD.

Therefore, microglia are seen as a key target for the development of new treatment approaches, with many emerging strategies to guide it towards a beneficial role upon neuroinflammation, by manipulating its metabolic pathways. In such a setting, many cellular targets in microglia that can be involved in phenotype modulation, such as membrane receptors, have been identified and pointed out as possible targets for further experiments and therapeutic approaches[7]. In this article, we review the major finds about the role of microglia in CJD, including its relationship to some of the risk factors associated with the development of the disease. Furthermore, considering its central role in neural immunity, we explore the microglial connection with other elements of the immune system and cell signalling, such as inflammasomes, the complement, and purinergic systems and their possible therapeutic targets in this disease.

## Epidemiology

CJD is the most common human form of transmissible spongiform encephalopathy (TSE). This group of pathologies is rare in humans, affecting approximately one person in every one million per year worldwide[8]. Beyond the CJD, other human TSEs include Gerstmann-Straussler-Scheinker disease (GSS), fatal familial insomnia (FFI) and kuru.

Among its three main categories, sporadic CJD is responsible for at least 85% of cases in the United States and worldwide, affecting men and women equally. The familial or hereditary CJD – in which mutations in the PRP gene lead to the development of the disease – is responsible for 5% to 10% of CJD cases in the United States. Acquired (iatrogenic) CJD – transmitted from contact with infectious nervous tissue or contaminated neurosurgical instruments – covers less than 1% of all cases of CJD[9].

Although it is a recognized type, with cases reported in several countries, the variant form of CJD (vCJD) does not comprise the main category. Its occurrence is due to the ingestion of beef infected with bovine spongiform encephalopathy (BSE). However, with the drop in the incidence of this disease in cattle and the dietary protection measures implemented, the occurrence of the variant form has become extremely rare[10].

## Clinical features

A typical CJD patient, which is frequently a patient affected by sCJD, usually starts manifesting the disease through non-specific neurological symptoms, such as psychiatric symptoms, memory deficits, and visual impairment. The clinical course progresses rapidly in the span of a few months into worsening cognitive dysfunction, ataxia, and myoclonus, finally reaching a state of akinetic mutism and death in less than one year. Patients who fit this description and manifest the alleged ‘classic triad’ – quick and gradual cognitive function decline, myoclonus, and periodic sharp wave complexes on electroencephalography – are considered classic CJD patients. When subjected to MRI, those patients usually manifest hyperintensity in the regions of the cerebral cortex and striatum[11].

The age onset of sCJD is usually 55 to 75 years old and patients will likely experience headache, fatigue, and sleep disturbances. Soon, they also may start complaining of memory deficits and numerous psychiatric symptoms like mood swings, agitation, irritability, depression, and apathy. Although less common, sensory symptoms like lack of coordination and visual impairment may also be present. As sCJD progress, confusion, disorientation, and deficits regarding planning and judgement abilities are manifested and become increasingly noticeable, along with myoclonus and muscle stiffness and twitching. Patients eventually completely lose mobility and ability to speak, entering a deteriorated state of coma[12].

Despite the so-called ‘typical’ clinical features of CJD mentioned in the last paragraph, this prion disease presents itself in significantly distinct ways [13,14], adding to the challenge of an early diagnosis of this condition. vCJD, for example, has a very specific pattern shown on MRI, different to the one mentioned previously for sCJD. vCJD patients show a symmetrical hyperintensity in the posterior thalamus, associated with the anterior putamen, called the ‘pulvinar sign’. This sign is reported to have a high sensitivity and 100% specificity for vCJD and can coexist with hyperintensity of the mediodorsal thalamic nucleus, in this case, displaying a hockey stick appearance, thus the ‘hockey stick sign’ denomination[14].

Another difference in the clinical features of vCJD, in comparison to sCJD, is that it affects younger patients, usually around their twenties. It usually starts with psychiatric symptoms, just like in sCJD, but also painful dysesthesias. Neurological signs do show up, but only later in the disease than what would be expected in sCJD. Prions have been detected in blood and urine samples of patients diagnosed with symptomatic vCJD and since the potential infectivity of those prions hasn’t been completely ruled out yet, extra caution should be practiced in situations of possible exposure to body fluids from vCJD patients[12].

The phenotypic heterogeneity of the disease became clear soon after Jakob’s description of the classic CJD when various reports appeared pointing to distinct patterns of clinical manifestations and distribution of pathological lesions that didn’t fit the classic model. Three CJD cases with distinct clinical features were reported by Heidenhain [15] in 1929. Comparing to the description of Jakob’s cases, these patients were characterized by shorter disease duration and two of them had cortical blindness, along with a more pronounced pathology in the occipital cortex. Moreover, spongiform degeneration was observed in two of these three cases. Later, Meyer et al. [16] concluded that there was a specific variant of CJD when they reported cases that showed prominent visual disturbances, for which they suggested the term amaurotic or Heidenhain variant (HvCJD)[15].

The clinical pattern can be classified as typical HvCJD when visual disorders occur as the leading symptom and remain the predominant manifestation during disease progression. As HvCJD affects the parieto-occipital cortex, cortical blindness is the classic clinical symptom. At disease onset, due to visual deterioration, with visual field restriction, blurred vision, vision loss, or even total blindness, patients not fully demented commonly give up reading or watching television.

Isolated visual symptoms such as poor vision, disturbed perception of colours or structures, visual defects, hemianopsia, visual agnosia, abnormal color/spatial perception, and optical distortions, as well as optical hallucinations without any ocular disease, may also occur. Other possible manifestations include metamorphopsia, optical hallucinations, or visual neglect[17].

## The complement system

In CJD and other transmissible spongiform encephalopathies, PrP^Sc^ accumulates in the CNS and secondary lymphoid tissues of infected hosts, but before that, they interact with the innate immune system at the initial site of infection, the peritoneal cavity. Thus, these proteins would deal with complement elements, dendritic cells, macrophages, and monocytes. Complement proteins are known to bind foreign bodies and altered self-particles, raising doubts about their role in CJD[18].

In this context, murine prion proteins, both from recombinant sources and diseased tissues, have been shown to activate the classical complement pathway. Studies have demonstrated that complement elements, such as C3 and C1q, play a role in prion neuroinvasion, since its depletion delays the development of the disease in mice, after intraperitoneal inoculation of a prion-limiting inoculum. In addition, it was observed that mice C1q-depleted have the same susceptibility to PrP^Sc^ infection by intracranial injection as wild-type mice. This all suggests a role of the complement system in the spread of prion infection from the intestine to the CNS [18–20].

A hypothesis suggests that PrP^Sc^ activates the complement system and becomes opsonized, leading to its recognition by the dendritic cells in the intestine, which acts in the dissemination of these proteins to the secondary lymphoid tissue, where they can be presented to follicular dendritic cells, which have complement receptor CR2 and PrP^C^ expressed in their plasma membrane, constituting a good environment for this PrP^C^ to become PrP^Sc^, contributing to the spread of the disease[18]. Kujala and colleagues demonstrate how PrP^Sc^ enters through Peyer’s patches primarily through specialized enterocytes, with facilitated entry by microfold cells (M cells), specialized for transepithelial transport of macromolecules and particles and collaborate to an appropriate immune system response [21]. However, in some pathogenic conditions, microorganisms use the M cells as a gate to the mucosal tissue, and studies *in vitro* have been shown that M cell-like cells transcytose scrapie agents [22,23].

Other study groups believe that small particles of prions can travel through the lymphatic system and enter lymph nodes through afferent lymphatic vessels, where they would encounter B cells. On the other hand, larger particles of prions or complement-prion complexes would be transported by monocytes. Macrophages would be able to capture large and small aggregates with or without complementary elements. These proteins would later be presented to follicular dendritic cells, where they would replicate[17].

In the CNS, the complement system would be involved in increasing microglial activation and improving the formation of complexes of PrP fibrils. Studies with stimulation of human microglia with synthetic peptides based on human and mouse prion proteins have shown that C1q enhances microglial activation, with more cytokine secretion (IL-6), but this effect is mainly observed and improved when C1q is co-incubated with serum amyloid P component (SAP). In addition, C1q, especially when associated with SAP, can promote the formation of fibrous PrP aggregates and accelerate this process, which improves the contribution to microglial activation[20].

Therefore, the complement system activation of human microglia by fibrillar prion would act in prion disease in its initial pathogenesis, promoting neuroinvasion after peripheral inoculation and improving microglial activation and the formation of PrP fibril complexes.

## Inflammasomes

Inflammasomes are cytosolic multiprotein complexes that function as intracellular sensors of pathogens or dangerous host-derived signals. They are central agents in the activation of immune responses to neurodegenerative diseases, stimulating the release of pro-inflammatory cytokines, such as interleukin IL-1β and IL-18[21]. IL-1β levels were elevated in the cerebrospinal fluid of patients with Creutzfeldt-Jakob Disease (CJD), which raises the importance of clarifying the role of inflammasomes in this pathology, as well as in other prion diseases [24,25].

Microglia acts as the main innate immune cells of the brain, being involved with the expression and activation of inflammasomes, through cytosolic pattern recognition receptors (PRRs), as members of the NLR and ALR families[21].

In summary, the stimulus for the formation of inflammasomes induces oligomerization of PRR proteins and recruitment of pre-existing peptides from pro-caspase-1, leading to their proximity-induced self-activation. NLRP3, the most well-known and studied inflammasome, responds to a wide spectrum of stimuli and is characterized by the cleavage of pro- IL-1β and pro-IL-18 to their mature forms by active caspases-1. In addition, caspase-1 can cleave gasdermin-D protein, releasing its N-terminal fragment, which translocates to the plasma membrane and induces pyroptosis – consequently, more inflammatory mediators are released[21].

Different views permeate discussion about the involvement of inflammasomes in prion diseases. Although high levels of IL-1β have been reported in the cerebrospinal fluid (CSF) of patients with Creutzfeldt-Jakob Disease [24,25] and in the brains of mice inoculated with PrP^Sc^ [26–29], others researches have shown that there was no significant difference in IL-1β levels in the brain of terminally ill rats inoculated with RML6 prions[27]. In addition, genetic ablation of NLRP3 did not demonstrate any significant changes in IL-1β levels at the terminal stages of CJD, which suggests that NLRP3 inflammasome does not play a significant role in its pathogenesis[21].

On the other hand, studies have shown that active inflammasomes, in addition to being pro-inflammatory, also cause a decline in the cell’s autophagic capacity[30]. Conversely, the stimulus to autophagy can also suppress inflammatory reactions, attenuating them through the degradation of pro- IL-1β [31–33]. In this context, the stimulus to autophagy can be used as an approach to control inflammation in prion diseases[33].

Autophagy is a highly regulated mechanism focused on the elimination of dysfunctional or unusable cellular components. This phenomenon, although physiologically present, is critically important in neurological conditions marked by inappropriate protein accumulation – such as Parkinson’s disease, Alzheimer’s disease, and prion diseases -, regulating the immune and inflammatory responses of these pathologies[34].

Research demonstrates that NLRP3 inflammasome, through active caspase-1, negatively regulates the cellular autophagic response to PrP106-126 stimulus in BV2 microglia. This suggests that inhibition of caspase-1 activation may increase the cell’s autophagic capacity, working as a possible effective therapeutic method in prion diseases[33].

It can be seen, therefore, that despite the interest around the role of inflammasomes in prion diseases, their contribution to the etiopathogenesis of these pathologies – including, Creuzfeldt-Jakob Disease – is not completely elucidated, requiring new approaches to clarify the current contradictions and allow the development of safe and effective therapeutic methods.

## Loss of homoeostatic functions in reactive microglia

It’s very common to see microglial activation being classified into two polar opposite processes: a classical one, which results in a proinflammatory phenotype, also referred to as M1; and an alternative form of activation, which results in an anti-inflammatory type of microglia (M2 microglia)[35]. Even though this M1/M2 model has been useful, especially regarding experiments on microglial behaviour in vitro, recent transcriptome studies have identified a more complex and diverse mechanism of activation that is greatly influenced by the environment surrounding microglia. Also, analysis of this process in vivo demonstrated that activated microglia rarely express themselves as M1 or M2. In fact, in models of neurodegeneration, these cells express both neuroprotective and neurotoxic factors[36], suggesting that there are more microglia phenotypes than the only 2 previously assumed and that the old debate about whether activated microglia are ‘good’ or ‘bad’ might not as important as it seemed to be since they can take on many different roles depending on context.

In normal conditions, microglia are found in the central nervous system expressing a homoeostatic transcriptome profile, constantly surveying the area for potential abnormalities, taking part in neurogenesis, synapse modulation, and development of cognitive functions [35,36]. To do that, they are influenced by cytokines and other substances surrounding them, as well as by their direct interaction with many of the cells present in the CNS. Neurons, for example, may deliver ‘on’ or ‘off’ signs that influence the activation of microglia[37]. In healthy brains, microglial activation is closely controlled by CD200-CD200R signalling[37]. CD200 is a protein part of the immunoglobulin superfamily, expressed on the neuronal membrane surface, whereas C200R – the CD200 receptor -, is mainly found in the macrophage lineage, including microglia. This signalling pathway has been identified as one of the critical pathways in diminishing microglial activation, as its disruption results in a quicker and more detrimental microglial response, resulting in neurodegeneration[37].

It has also been hypothesized that a favourable or harmful reaction of the local microglia response – part of the innate immune response – depends on a finely measured communication between innate and adaptive immune constituents, usually T cells that can cross the blood–brain barrier and impact microglial function[37]. Recent studies have additionally pointed out a shift in microglia function and shape towards a detrimental phenotype that seems to be directly associated with senescence [38]. The most distinctive feature of the aged microglia is their dystrophic state, with loss and shortening of their cytoplasmatic processes, cytoplasmic spheroid formation, and myelin fragmentation. They are widely expressed in the human brain of older subjects and exhibit a disrupted inflammatory behaviour and a neurotoxic state in response to activation[37]. Studies in animal models also concluded that dystrophic microglia, in the brain of older rats, had a significantly higher proliferation rate after neuronal injury in comparison to microglia response in young rats[37]. It is accepted, thereby, that with ageing, a progressive increase in activation and proinflammatory cytokine secretion by microglia creates an antineurogenic microenvironment which lessens neural stem cell proliferation[36].

Microglia that lose their physiological homoeostatic state is also referred to as disease-associated microglia (DAM). The emergence of this phenotype is promoted by numerous factors, such as exposure to apoptotic bodies of neurons, lipid degradation products, protein aggregates, and myelin debris[39]. According to the more recent data, the phagocytic activity of microglia in that pathological state depends on the activation of TREM2, a receptor expressed on myeloid cells and osteoclasts. TREM2 can bind to ligands like LPS, lipids, and lipoproteins, inducing the expression of multiple genes responsible for phagocytosis and rearrangement of the cytoskeleton[39]. Genetic polymorphisms in TREM2 are associated with an increased risk for neurodegenerative disease, most notably Alzheimer’s disease. An essential aspect that is yet to be understood is the multiple roles of TREM2 on modulating microglial activity, since the absence of TREM2 may be protective in some instances and intensify neuronal damage in others[40].

An independent study performed transcriptomes of isolated microglia in conditions, such as ageing, mouse models of AD (APP/PS1), ALS (SOD1G93A), and multiple sclerosis[36]. A similar microglia molecular response was found in the four different models, with loss of homoeostatic genes like P2ry12, Tmem119, Gpr34, Csf1r, Hexb, and Mertk, along with upregulation of inflammatory genes like Axl, Clec7a, Ccl2, and Apoe. This microglia phenotype was named microglia neurodegenerative phenotype (MGnD), but it has many similarities to DAM and could be considered a subtype of DAM (the existence of subclasses has been supported by weighted gene correlation network analysis). Microglial Apoe upregulation was associated with disease progression in three out of the four models, suggesting that DAM might take up a neurotoxic role. Also, the study concluded that Apoe deletion significantly prevented overexpression of DAM genes, such as Clec7a, Gpnmb, and Lgals3 and that targeting genes related to TREM2 kept microglia from losing their homoeostatic abilities and manifesting themselves as DAM[36].

**Figure 1.**
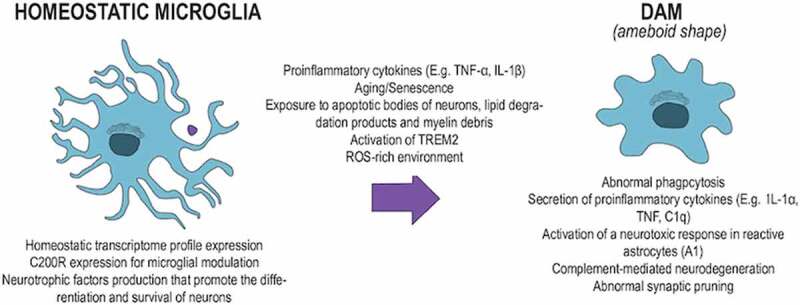
The homoeostatic microglia turning into an ameboid shape after pro-inflammatory stimulation

An active microglia’s shape and motion may reveal some characteristics about their profile of response to stimuli. Significant injury of neuronal tissue induces microglia to transform from a normal ramified shape to an amoeboid one, simulated in Figure 1. In this scenario, cell bodies become enlarged and cell processes are shortened, covering less area than they used to[36]. This amoeboid shape indicates a highly activated state related to more prominent phagocytosis and proinflammatory function. These kinds of microglia also have their proinflammatory states promoted and sustained by various receptors expressed by them[36]. The purinergic receptor P2X7, in response to elevated ATP concentrations, prompts the microglial release of TNF-α. Adenosine receptors, like A2a, promote the release of inflammatory mediators and phagocytosis. Receptors for neuropeptides, like substance P and bradykinin, make the transition of microglia into an inflammatory state an easier process and consequently, they can magnify inflammation response[36].

Under reactive oxygen species (ROS) production conditions, stimulation of toll-like receptors (TLR) can trigger the activation of NADPH oxidases (NOX)[40]. The detrimental role of activated NOX2 has been verified in multiple experiments involving distinct neurodegenerative diseases[40]. AD subjects presented activated NOX2 within microglia, culminating in the release of ROS, along with consequent neurotoxicity[37]. In prion diseases, microglia can clear the CNS of PrP^Sc^ with the collaboration of astrocytes, which release lactadherin (MFGE8), a protein that tags PrP^Sc^ -containing apoptotic cells for phagocytosis[40]. Shortage of MFGE8 results in less effective removal of cerebellar apoptotic bodies and augments the number of prions, promoting a more accelerated progression of disease in prion-infected mice models[40].

Exposition of microglia to different stimuli including LPS, ATP, arachidonate, and cytokines like TNFα and IL-1β produces extracellular NO. This prompts a consecutive stimulation of NOX, which promotes a fast cleavage of extracellular NO, as well as peroxynitrite formation[40]. These reactions are potentially neurotoxic and because of that, the combined actions of iNOS and NOX seem to be of great importance in matters of a neurodegenerative response of microglia[40]. In general, the inflammatory response – not just in this case but in most scenarios involving detrimental active microglia – results in the activation of transcription factors, such as NF-κB, activator protein-1 (AP-1), cAMP response element-binding protein, CCAAT/enhancer-binding protein, and IRF, which correlates to an intricate mechanism of transcriptional regulation of cytokines[41].

Experiments on neurodegenerative disease models suggest that microglia express themselves in beneficial or harmful ways depending on the timing of their activation regarding the stage of the disease[40]. Therefore, the timing of microglial activation could be decisive in the maintenance or not of homoeostatic functions of microglia. CX3CR1 is a specific marker of microglia whose function in modulating microglial activity is also a subject of studies. CX3CR1-mutant animals showed a temporary decrease in microglial cell quantity, which was associated with an increase in density of hippocampal CA1 neurons and a decline in functional connectivity through the CNS[35]. This happened as a result of an accumulation of immature synapsis due to insufficient microglia to exercise its function of synaptic pruning, which could improve the efficiency of those synaptic connections.

It’s worth noting that microglia are the primary cells in the brain that express many essential complement components. Under normal CNS conditions, complement proteins not only target pathogens for immune combating but they are also involved in synapse pruning, disease-associated synapse loss, and cognitive deterioration related to ageing[35]. Ageing, specifically, is correlated to a significant boost in the microglial expression of C1q, C3 synapse tagging, and elimination[35]. In this case, there is an exaggerated activity of microglia on synapse modulation, which relates to abnormal microglial engulfment of synapses via CR3 and complement-mediated neurodegeneration, with particular brain regions affected and behavioural impairments being disease-specific[35].

Activated microglia can phagocytose dead, damaged, and even normal cells in the human brain, commonly through the production of reactive oxygen species (ROS)[35]. Part of this phagocytic mechanism is promoted by microglial expression of the TYRO3, AXL, and MER (TAM) receptor tyrosine kinases and their ligands[35]. It is worth noting that AXL expression is upregulated in many neurodegenerative disease models, proposing a connection between abnormal microglia phagocytosis and neurodegenerative disease development[42]. In these same kinds of pathologies, other results of microglial activation have also gained attention. It seems that microglia–astrocyte interaction may help protect against microorganism infections, but when activated in other contexts, this mutual interaction may help propel neurodegeneration several diseases, including Alzheimer’s disease, Parkinson disease, and amyotrophic lateral sclerosis (ALS)[35].

It has been recognized that microglia activated by injury secrete IL-1α, tumour necrosis factor, and C1q – proinflammatory cytokines and a complement component that are sufficient to induce a neurotoxic response in reactive astrocytes (A1)[35]. These astrocytes are predominant in neurodegenerative disease and are characterized by a loss of physiological phagocytic and synaptogenic functions, as well as a gain of cytotoxic abilities and a promotion of upregulation of C3 and other components constituents[35]. It has also been determined that the development and activation of microglia can be influenced by some not so obvious factors, such as microbiota and maternal-induced immune activation[38], which may further promote the development of neuropathologies.

Ultimately, it’s important to recognize that most of the studies on microglial roles in disease have mice as experimental models, which might not reflect truthful enough insight on microglial roles. Many observations point out that rodents and human microglial are distinct; *in vitro* microglia collected out of rats proliferate at a considerably higher rate than human microglia, for example[40]. There are also distinctions regarding neuroinflammation mediators, as was noted that some of them are quite important in mice but of minor relevance in adult human microglia, like TGF-β[40]. Also, the LPS receptor TLR4 was found to be an important approach to activate rat microglia, but it doesn’t seem as important in humans[40]. Another major substance of microglial activation, the synthesis of NO through the activity of inducible NO synthase (iNOS) is a potentially controversial aspect to be studied on rats, as their microglia respond more expressively with greater production of NO upon inflammatory stimuli than human microglia[40].

## CJD Risk factors (TREM2?)

Neurodegenerative disorders share common pathological features that revolve around an inflammatory process. This justifies the search for common risk factors and pathological mechanisms between these disorders. In the nervous system, the triggering receptor expressed in myeloid cells 2 (TREM2) is expressed mainly in microglia and is related to the phagocytic function of these cells. Since microglia are the central immune cells of the CNS and TREM2 has been already related to Alzheimer’s disease, frontotemporal lobar degeneration, Parkinson’s disease, and others, it is questioned whether this receptor is also involved in prion disorders, like CJD[41].

Studies with models of mice with the prion disease showed that the expression of TREM 2 in microglia was increased by prion infection, but prion-infected TREM2^−/−^ mice have reduced microglial activation[41].

Other studies have shown that TREM2 knockout mice have upregulated genes, such as Sall1 and TGF-β1, responsible for maintaining the resting state of the microglia, supporting the idea of TREM2 as a receptor that promotes microglial activation in inflammatory circumstances. In addition, microglia with a lack of TREM2 have a reduced response power of migration to chemotactic signals from injured and dying tissues, as well as a deficiency in the outgrowth of microglia processes [41,43,44].

However, when viewed from the perspective of prion disease, TREM2 did not show a contribution to its pathogenesis: there were no significant differences in the disease incubation time or prion titres between the TREM2^−/−^, TREM2^−/+^ and TREM2^+/+^ mice groups, after intracerebral inoculation of Rocky Mountain Laboratories PrP^Sc^ strain (RML6). Also, a study of the relationship between the presence of R47H TREM2 variant – already related to Alzheimer Disease, and an increased risk for sporadic CJD, showed no altered risk for these patients [45,46].

Therefore, TREM2 is seen as a key feature of the microglial response to neurotoxic lesions, thus having a role in neural response to infection by prions, but there are still no consistent findings that directly relate it to prion disease.

## Other CJD risk factors

Recently, a study identified GMR8, a gene that encodes mGlur8 – a protein of the family of metabotropic glutamate receptors -, as a potent risk factor for CJD, being related to the transduction of physiological and cytotoxic signals mediated by PrPC. In this context, carriers of a risk allele in rs6951643 – a variant located in an intronic region of the GRM8 gene – tend to have an increase in mGlur8 expression in microglial cells compared to non-carriers, representing a potent marker for disease risk mapping[46].

Sporadic Creutzfeldt-Jakob disease (sCJD) can be divided – based on the biochemical characteristics of the prion protein and the allelic variation of the codon 129 of the prion protein gene (PRNP) – into 6 subtypes, of which MM1 and VV2 are the most common. The PRNP gene plays a central role in the degree of disease susceptibility[45], with the polymorphism encoding methionine (M) or valine (V) at codon 129 (PRNP M129V) registered as a risk factor[47].

Also, polymorphisms in certain genes related to inflammation have been identified as risk factors in neurodegenerative diseases associated with abnormal protein aggregation (genetic and transcriptomic profiles). A study analysed the gene expression of cytokines and mediators of the immune response in the area 8 of the frontal cortex and in the cerebellum (both post-mortem) of patients with sCJD – with cases of subtypes MM1 and VV2 – compared to a control group. The results showed differences in the regulation of gene expression, depending on both the region observed and the subtype of the pathology – the genes were overloaded in the area 8 of the frontal cortex in sCJD MM1 and in the cerebellum in sCJD VV2[48].

The unregulated expression of these genes – including pro and anti-inflammatory cytokines, toll-like receptors, colony-stimulating factors, cathepsins, members of the complement system, and members of the integrin and CTL/CTLD family – has been verified in neurons and other glial cells, mainly microglia, suggesting an important interaction between these cells in modulating inflammatory response[49].

Thus, the complex scenario that permeates neuroinflammation in sCJD stands out, requiring further studies to understand its pathogenesis and identify new therapeutic targets.

## Microglial targets in CJD

Microglia is a key component of the CNS immune system and is capable of triggering both inflammatory and anti-inflammatory responses. When activated, they act as cellular initiators of the innate and then adaptative brain immune response. Although the specific mechanisms are not completely elucidated, an inflammatory process dominated by microglia seems to be an important feature of the immune response of the CNS in CJD[49].

The inflammatory response is deeply associated with the pathogenesis of CJD and can be identified as an early event of the disease, as shown in a study that investigated inflammation and apoptosis markers on the brain and CSF of CJD patients[24]. In this study, inflammatory response mediated by microglia, astrocytes, and IL-1 was observed, showing a significant correlation between activated microglia and the presence of IL-1, which was found at increased levels in CJD patients. This pattern was not dependent on the stage of the disease, suggesting that microglia, which produces IL-1, is early activated, and remains activated throughout the disease progression[24]. Targeting the microglia-mediated immune response could be important to define novel therapeutic approaches and to modify the disease course[50].

Microglial activation precedes neuronal damage, and increases with the disease progression, acting as a major cellular inflammatory mediator in prion diseases, although its exact role is yet to be elucidated. However, it isn’t clear if the activation happens directly by the accumulating misfolded PrP^Sc^ or because of synaptic damage[48]. Activated microglia found in the brain of CJD patients present spatial correlation to PrP^Sc^ depositions. Nonetheless, PrP alone seems not to induce microglial inflammatory response [49,50]. Brandner et al. [51] demonstrated that early stages of the disease in the graft were characterized by spongiosis, gliosis, and reduced synaptophysin immunoreactivity, which was found in terminally sick tga20 mice, whereas intermediate-stage grafts showed status spongiosis with dramatic ballooning and loss of neurons, gliosis, and stripping of neuronal processes.

Activated microglia release cytokines that induce apoptosis and inflammation processes. In CJD, neuronal loss is mainly related to apoptosis, and the important role of microglia in this process is supported by the significant correlation found between the number of microglia and areas of severe neuronal damage[24].

The activation process is very dynamic and complex and involves many variants. When these cells are modified from a resting to an activated state, they can transform into different phenotypes, including a proliferation stage, migratory stage, homing, and motility stage, and phagocytic stage[48]. Microglia can switch from a dominant anti-inflammatory and regenerative phenotype, driven by anti-inflammatory cytokines like IL-4 and IL13, immune complexes, IL- 1 R ligands, IL-10, TGF-β, and glucocorticoids, to dominant pro-inflammatory and neurotoxic phenotype, induced by (IL)-1β, the toll-like-receptor (TLR)-4 agonist lipopolysaccharide (LPS), the TLR-3 agonist Poly I: C, and tumour necrosis factor-α (TNFα), along with the disease progression [6,50].

The potentially harmful effect of microglial activation in the tissue is likely correlated to the phenotype of the cells and not simply to the presence on the brain of microglia itself. Therefore, microglia assume a dual role in pathogenesis, acting both as a protection and as an inflammation and disease progression factor, depending on the different dynamics and molecular pathways of cell activation [49,52].

This is supported by previous studies that demonstrated that in the absence or deficiency of microglial cells, there is no change in the course of the disease, or the neurodegeneration process is accelerated, which points to a protective function of these cells on CJD [49,50].

Phagocytic microglia clear apoptotic neurons and debris and are characterized by the lack of IL-1. Studies have shown that phagocytic activity is a protective mechanism, considering that the inhibition of this function leads to a more rapid progression of the disease and an increase of PrP accumulation[49]. Phagocytosis by microglia is dependent on milk fat globule epidermal growth factor 8 (MFGE8). Without MFGE8, pathology progression occurs more rapidly[49].

In prion disease, the activation of CSF1R and the transcription factors PU.1 and CCAAT/enhancer-binding protein alpha (C/EBPα) modulate microglial proliferation[49]. The survival of microglia in the CNS is dependent on consistent signalling through a tyrosine kinase receptor called CSF-1 R. The ligands to this receptor are CSF-1 and IL-34, which are produced and secreted predominately by astrocytes and neurons. Studies using a CSF-1 R inhibitor showed an increase in survival of infected mice, lower microglia proliferation in the hippocampus and thalamus, and a slower disease progression and neurodegeneration[53]. Other than the decrease in the microglia population, these benefits are correlated to a higher expression of genes associated with the M2 phenotype and a decrease of genes associated with the M1 phenotype. M1 phenotype is linked to a protective function of the microglia and M2 seems to contribute to neurodegeneration[52]. These findings suggest that selective and specific control of microglia proliferation is needed to control disease progression and to completely understand microglial role in CJD[49].

Signal transduction pathways are also modulated during the pathology progress. In prion disease models, activation of the signal transducer and activator of transcription (STAT)- and Nuclear factor kappa-light-chain-enhancer of activated B cells (NFkB) pathways have been observed, correlated to the up-regulation of STAT- and NFkB-responsive genes, including many cytokines and chemokines, as well as by the detection of increased phosphorylation of STAT1 and STAT3 specifically[49]. IL-10, a pro-regenerative microglia marker, inhibits cytokine-activated Janus kinase (JAK)/STAT-1 signalling, thus exerting an anti-inflammatory influence on microglia. IL-10 release can be enhanced through the expression of the metabolic enzyme Arg1 in microglia, which is induced by the melanocortins α-MSH and NDP-MSH actions on G protein-coupled receptors (GPCRs)[6].

IL-4 and IL-13 exert influence on a series of molecular pathways that impact gene expression and cellular metabolism towards an anti-inflammatory pattern, consequently affecting microglia reprogramming[6].

On the prion-disease brain, there is up-regulation of both pro- and anti-inflammatory factors. The anti-inflammatory spectrum is dominated by the expression of transforming growth factor β (TGFβ), C–C Motif Chemokine Ligand 2 (CCL2), and prostaglandin E2 (PGE2). The pro-inflammatory response includes, among other factors, IL-1β and tumour necrosis factor α (TNFα). However, reduced levels of CCL2 and PGE2 do not significantly affect the disease progression. Contrariwise, the lack of TGFβ leads to faster neurodegeneration, which demonstrates its essential role in the CNS immune response[49]. Anti- and pro-inflammatory microglial targets are described in Table 1.

**Table 1.** Pro and anti-inflammatoy microglial targets in CDJ

Pro-inflammatory	IL-1 (IL)-1β Toll-like-receptor (TLR)-4 agonist lipopolysaccharide (LPS) TLR-3 agonist Poly I: C Tumour necrosis factor-α (TNFα) CSF-1 R STAT and NFkβ signiling pathways
Anti-inflammatory	IL-4 IL-13 Immune complexes IL- 1 R ligands IL-10 TGF-β Glucocorticoids Endocannabinoid type 2 receptor (CB2) Histamine receptors Nuclear receptors PPARs-y Trk-A receptors C–C Motif Chemokine Ligand 2 (CCL2) Prostaglandin E2 (PGE2) miRNA-146a

Suppression of microglial IL-1 receptor type 1 delays disease onset and slows down pathological progression[49].

In general, microglia express different types of receptors and channels that can induce microglial reprogramming towards anti-inflammatory phenotypes. One of these receptors is the histamine receptors and all four types are expressed by microglia. When activated, these receptors lead to a signalling pathway that increases anti-inflammatory genes and decrease expression of pro-inflammatory transcription factor NF-kB and NADPH oxidase 2 (NOX2) [6], which is up-regulated in the affected brain of CJD patients[49]. Using the same mechanism, the endocannabinoid type 2 receptor (CB2) can lead to sustained ERK1/2 phosphorylation, hence pNF-κB downregulation[6].

Mitochondrial energetic state and glucose and fatty acid metabolism pathways may be involved in microglial inflammatory modulation. Novel evidence suggests that at least p-NF-κB, iNOS, and NOX2 activity are influenced by the bioenergetics state of microglia[6].

Nicotinic receptors can induce a pro-regeneration stage of microglia by inducing upregulation of antioxidant genes. It also promotes reduced expression of p-NF-κB and its p65 subunit and inhibits p38 mitogen-activated protein kinase (p38-MAPK), elements that play an important role in inflammatory processes[6].

The activation of Trk-A receptors by the classical neurotrophin NGF can potentially revert a pro-inflammatory stage of the cell and drive it towards an anti-inflammatory phenotype, having a protective effect upon neurons by controlling microglia-neuron interaction. It also regulates microglial activities such as cytokine/chemokine secretion, motility, phagocytosis, and degradative pathways[6].

Nuclear receptors PPARs-γ play a vast role in the regulation of microglial activation and inflammation through many different mechanisms. Some of them include increasing anti-inflammatory genes Arg-1 and IL-4 expression, promoting microglial phagocytic function, reducing activation of STAT-1 and NF-κB, and metabolic reprogramming[6].

MicroRNAs (miRNAs) act as key regulators during neural and immune cell differentiation and activation. Various types of miRNAs respond differently to pro- or anti-inflammatory stimuli. Specifically, miRNA-146a seems to have a protective role in influencing the microglial activation state by enhancing mitochondrial energy metabolism and fatty acid oxidation[6]. It can also play a role in immune response in prion disease by suppressing pro-inflammatory proteins interleukin-1 receptor-associated kinase 1 (IRAK1) and TNF receptor-associated factor-6 (TRAF-6) [6,49].

Microglia activation mechanisms and metabolic pathways are not completely elucidated, but they are certainly important and determinant on the immune response and disease progression in prion diseases. As microglia plays an essential role in the pathogenesis of CJD, many microglial targets can be key points to therapeutic approaches and a better understanding of the disease features.

## Purinergic signalling

Adenine nucleotides, such as ATP, have a known role in signalling physiological and pathological processes, is considered one of the main molecules of ‘damage-associated molecular pattern’ (DAMP)[54]. In the CNS, they are released from astrocytes and neurons as a result of neuronal damage and remain in high levels in the peritraumatic region after injury. It has been shown that microglia responds to the rapid increase in ATP levels immediately after neuronal damage, through purinergic receptors, by releasing cytokines, projecting their processes, and migrating to the site of injury[55]. Experiments using laser-induced injury within the cortex of transgenic mice with and without the application of apyrase – an ATPase that hydrolyzes both extracellular ATP and ADP – shows that extracellular ATP and activation of P2Y may even be necessary for the rapid microglial response, suggesting that ATP could be the main chemoattractant responsible for directional extension of microglial processes towards the site of damage[55]. Purinergic system has been shown to be critical in several degenerative diseases involving neuroinflammation, such as Alzheimer’s Disease, Multiple Sclerosis, Amyotrophic Lateral Sclerosis, and also to control microglial functions during neuronal infection with the neurotropic virus [56–58].

With this knowledge, it is reasonable to think that purinergic signalling may play a key role in microglial activation upon prion infection. It was demonstrated by comparing PrP^Sc^ -infected and non-infected microglia, that sensitivity, and expression of the P2X7 receptor (P2X7R) were increased in infected cells, leading to higher intracellular Ca2+ concentration, formation of more pores in the cell membrane, induction of microglial death, increased release of IL-1β and upregulation of P2X7R mRNA. Also, sensitivity and expression of P2X7R returned to normal levels in self-cured microglia, supporting a possible critical role of these receptors in prion disease[59].

Although the current knowledge about the relationship between microglia and purinergic signalling leads to an important role of the purinergic system in CJD, there is a lack of knowledge on the subject and more studies must be done, to elucidate more related receptors and to detail-related cell processes.

## Microglia in Creutzfeldt-Jakob disease: current hot topics

Microglia’s importance in CJD and the fact that it can influence disease progression are widely accepted. However, the specific molecular pathways that lead microglia either to a protective or to a harmful phenotype remain unexplained. The mechanisms involved in the cellular interactions that contribute to neurodegeneration or neuroprotection are also not completely clear and can be a significant target to new therapeutic approaches.

When microglia are activated by different stimuli, distinct cytokine patterns can be observed. An increased expression of IL-1 and TNF-α, for example, points to a pro-inflammatory regulation, while higher levels of TGF-β, IL-4, and IL-10 are linked to a pro-regenerative anti-inflammatory regulation in mice [60]. Therefore, understanding how these processes work could help to target active microglia modulation through controlling cytokine expression on the infected tissue and consequently, possibly delaying neurodegeneration[5].

**Figure 2.**
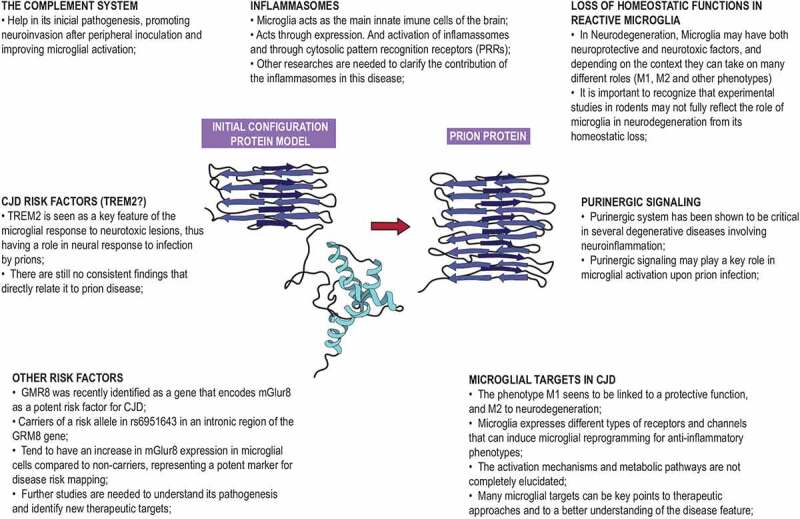
Main immunological features and possible therapeutic targets [61]

Recent studies have shown that many microglial receptors, such as CSF1R, PPARs-γ, and Tka-A, when activated or inhibited, can influence the phenotype of activated microglia in prion disease. Distinct ligands can also provoke different interactions and lead the cells into various molecular pathways. As an example, experiments using CSF-1 inhibitors have shown slower neurodegeneration and an increase in microglial anti-inflammatory phenotype expression[6]. Some features of the article are summarized in Figure 2.

Many experiments have shown, however, controversial, or inconclusive results. Thus, further, and continued investigation is needed to elucidate the dynamic mechanisms that modulate microglial activation in CJD to explore the already-existing promising therapeutic targets that can influence the prognosis of the disease and to create possible new treatment strategies.

## Possible therapeutic approaches

Until recently, pharmacotherapy of Creutzfeldt-Jakob disease was inconceivable. Therefore, treatment was limited to supportive and symptomatic care, with no feasible options to cure or reduce the progression of the disease[62]. Part of the problem in achieving better therapeutic alternatives is the lack of early diagnosis and more specific and objective follow-up criteria to monitor CJD’s progression and possible therapeutic benefits[63].

With emerging evidence highlighting the contribution of microglial expansion and activation to the pathogenesis of prion disease, targeting the microglia-mediated immune response, as well as its proliferation, seems to be a favourable option to interfere with disease progression[50]. Despite that, defining more precisely the mechanisms of neuroinflammatory processes in CJD remains a challenge, but it represents a crucial step in developing new and more effective therapeutic strategies.

Targeting microglial expansion through inhibition of CSF-1 R – a tyrosine-kinase receptor involved with their survival and proliferation – has been discussed in recent years as a potential therapeutic approach. However, results of animal experiments done so far have been controversial, with some of them showing improvement in clinical signs of prion disease and reduced synaptic degradation [50,51], while others found significantly accelerated prion disease progression, astrogliosis, and spongiform changes[50].

The mixed results shine a light on the different phenotypes of microglia and their distinct roles in the progression of CJD. More investigation is needed on discovering how to selectively target the specific pro-inflammatory microglia, so this can be a truly beneficial therapy. This is corroborated by the observation that more embracing approaches on eliminating microglia in animal models with prion disease had the worst outcomes [50,51]. The use of Ara-C (cytosine arabinoside), a non-specific blocker of mitosis, for example, promoted such a significant shift in microglial phenotype towards a pro-inflammatory state, that even with overall reduction of microglia, the mice experienced accelerated neuronal death[50].

Targeting the immune pathways which are dysregulated in prion disease has also had mixed results among recent studies. High levels of Prostaglandin E2 (PGE2), which correlates to microglial activation in prion diseases, have been found for more than two decades in the cerebral spinal fluid (CSF) of CJD patients[64]. The amount of PGE2 had a direct link with shorter survival times for those patients (despite its association with an anti-inflammatory cytokine profile)[64], and because of that, therapies that limited PGE2 concentration on the brain of CJD patients was tested.

Experiments with mice inoculated intracerebrally with CJD were made using indomethacin and dapsone, both inhibitors of PGE2 production, but only dapsone showed promising results regarding the delay of CJD progression [51,65]. Aside from that, other studies that came out, later on, showed no impact of the use of dapsone on prion disease progression[50], pointing out to the fact that the exact inflammatory mediators involved with microglial activation in CJD disease, their pathophysiological roles, and their relationship among themselves need to be further elucidated in order to come up with a more reliable therapeutic alternative.

Statins have been studied for the treatment of CJD initially because of their action destabilizing ‘lipid rafts’ – cholesterol-rich detergent-resistant membrane domains – that anchor PrP^C^ to the cellular membrane, allowing their conversion and replication into PrP^Sc^ [65]. But another seemingly important contribution of these drugs to the treatment of prion diseases is that they demonstrated an ability to lessen inflammation in multiple models of neurodegenerative disease[51]. In murine models of Alzheimer’s disease, atorvastatin limited the production of proinflammatory cytokines and lowered the number of microglia in the hippocampus[51]. Similar results were found in studies involving animal models of multiple sclerosis and Parkinson’s disease[51].

Again, just like other targets still under investigation to treat CJD, the real efficacy of statins on reducing neurodegeneration and neuroinflammation, analysed in human clinical trials of Alzheimer’s and Parkinson’s disease patients, was controversial. There were contrasting results regarding the reduction or not of symptoms, disease progression, and improvement of neurological functions in those patients, leading to a conclusion that they might not offer enough benefit[51].

Statin treatment was also tested in infectious prion protein mice models, showing little or modest statistically significant improvements in survival times[51]. A blinded protocol study involving mice with prion disease investigated the ability of simvastatin, pravastatin and atorvastatin to decrease neuroinflammation and improve survival, but gliosis and PrP^Sc^ deposition in the CNS were similar in treated and untreated mice[51]. Also, survival time remained unaltered, indicating that none of the statins tested was effective in reducing PrP^Sc^-induced neuroinflammation or neuropathogenesis[51].

Another therapeutic option would be targeting specific P2 purinergic receptor subtypes involved in microglial activation and recruitment to areas of neuronal damage[64]. Partial antagonization of those receptors could be executed so that less detrimental inflammatory mediators would be synthesized in the location of neuronal injury. Since those receptors, like microglia, mediate many different actions that could be either good or bad to the environment of neuronal damage, agonizing other subtypes of P2 receptors could also work as a CJD therapy.

On this note, essential roles, such as increasing production of neurotrophins, which would promote neuron survival, are also executed by P2 receptors[66]. Consequently, agonizing those specific P2 receptors might also be a potential option to treat prion diseases like CJD. Further studies to fully elucidate the role of each different variation of P2 receptors regarding microglia are fundamental before attempting to target these receptors in animal models. Another issue about P2 receptors, in general, is the lack of known and available highly selective ligands, which would allow better differentiation among specific receptor subtypes [66,67].

There are extra metabolic pathways and currently available drugs that could be explored to induce metabolic changes in microglia, shifting their activity towards the regeneration of neuronal tissue, but none of them tested yet on CJD models. Dichloroacetate (DCA), a drug approved for the treatment of multiple sclerosis (MS), among other disorders, has been shown to positively influence the expression of pro-regenerative microglia phenotype both in vitro and in a peripheral inflammation model[6]. Dimethylfumarate (DMF), another approved drug for MS, is also acknowledged as a means to induce the transformation of pro-inflammatory microglia into a neuroprotective phenotype[6]. Both could be potentially beneficial in prion diseases such as CJD.

Metformin, a widely used and known antidiabetic drug, has also been discovered as an agent that promotes a regenerative microglial phenotype, as well as a drug that significantly improved neurobehavioral function after situations of neuronal damage like stroke and traumatic brain injury, hinting that it may be used to intensify pro-regenerative microglial activity through AMPK activation and subsequent stimulation of oxidative metabolism[6].

PPAR-γ activation, which can be achieved through another class of antidiabetic drugs, is another way to force oxidative metabolism in microglia, especially through inhibition of the TREM2 by apolipoprotein E[39]. This signalling pathway would drive microglia into a neurodegenerative phenotype, as well as suppress some of their primary homoeostatic genes[39]. Thiazolidinediones, PPAR-γ agonists, have been demonstrated as a feasible alternative to reduce neuroinflammation in different models of brain diseases and to slow down neurodegeneration in patients with less severe dementia[6].

The protective microglial function may be also induced by inhibitors of aldose reductase (AR)[6], a metabolic enzyme involved in the polyol pathway – a two-step chemical process that is implicated in inflammation-related diseases, such as diabetes. FMHM, a type AR inhibitor, showed good results in experiments in vivo and in vitro, preventing the expression of inflammatory genes in microglia[6]. This is explained by the fact that it suppresses the activity of the phospholipase C/protein kinase C signalling, culminating in downstream inactivation of the NF-κB inflammatory pathway[6]. Two other AR inhibitors have been identified (Sorbinil and Zopolrestat), which also prevented the expression of a proinflammatory cytokine profile in similar ways, interfering negatively with NF-κB and MAPK signalling pathways in Aβ-treated microglia[6].

Up until now, what we do know for certain is that not all microglia respond in the same way, even to the same stimulus, and microglial actions are designed in a way that is particular to the context those cells find themselves in. Establishing what exactly the elements involved in that context are and making clear their relationship with microglia is fundamental before coming up with a strategy to control neuroinflammation and neurodegeneration via microglial targeting[37]. Given that microglia might assume contrasting roles, even neuroprotective ones, trying to inhibit all microglia without differentiating their function in a context-dependent manner should be given up[37]. In addition to that, our knowledge about microglial functions in CNS disease is acquired primarily through studying animal models, which may not accurately represent human conditions[35]. In this degree, until human clinical trials become feasible, there’s not much that can be assured regarding the topic.

## LIST OF ABREVIATIONS

CJD – Creutzfeldt-Jakob disease

CNS – Central Nervous System

sCJD – Sporadic Creutzfeldt-Jakob disease

PrP^C^ – Cellular prion protein

PrP^Sc^ - Infectious prion proteins

M2 – Anti-inflammatory profile

M1 – Proinflammatory archetype

TSE - Transmissible spongiform encephalopathy

GSS - Gerstmann-Straussler-Scheinker disease

FFI - Fatal familial insomnia

hCJD - Hereditary Creutzfeldt-Jakob disease

aCJD – Acquired Creutzfeldt-Jakob disease

BSE – Bovine spongiform encephalopathy

vCJD – Variant Creutzfeldt-Jakob disease

HvCJD – Heidenhain Creutzfeldt-Jakob disease variant

SAP – Serum amyloid P componentPRRs - Pattern recognition receptors

CSF - Cerebrospinal fluid

DAM - Disease-associated microglia

MGnD - Microglia neurodegenerative phenotype

ROS - Reactive oxygen species

TLR - Toll-like receptors

NOX - NADPH oxidases

MFGE8 - Milk fat globule epidermal growth factor 8

AP-1 - Activator

- Amyotrophic lateral sclerosis

A1 - Reactive astrocytes

TREM2 - Triggering receptor expressed in myeloid cells 2

RML6 - Rocky Mountain Laboratories scrapie strain

PRNP - Prion protein gene

M – Methionine

V – Valine

LPS – Lipopolysaccharide

TNFα - Tumour necrosis factor-α

C/EBPα - CCAAT/enhancer-binding protein alpha

STAT - Signal transducer and activator of transcription

NFkB - Nuclear factor kappa-light-chain-enhancer of activated B cells

JAK - Cytokine-activated Janus kinase

GPCRs - G protein-coupled receptors

TGF-β - Transforming growth factor β

CCL2 - C-C Motif Chemokine Ligand 2

PGE2 - Prostaglandin E2

NOX2 - NADPH oxidase 2

CB2 - Endocannabinoid type 2 receptor

p38-MAPK - p38 mitogen-activated protein kinase

miRNAs – MicroRNAs

TRAF-6 - TNF receptor associated factor-6

DAMP - Damage-associated molecular pattern

P2X7R - P2X7 receptor

DCA – Dichloroacetate

MS - Multiple sclerosis

DMF – Dimethylfumarate

AR - Aldose reductase
